# The Clinical Aspects of Arterial Disease

**Published:** 1909-07-24

**Authors:** T. Clifford Allbutt

**Affiliations:** Regius Professor of Medicine in the University of Cambridge


### Hospital Clinics.

### THE CLINICAL ASPECTS OF ARTERIAL DISEASE.

By SIR T. CLIFFORD ALLBUTT, K.C.B., F.R.S., M.D., D.Sc; Regius Professor of Medicine in the University of Cambridge.

(An address delivered at a meeting of the Metropolitan Branch of the British Medical Association, held at the Criterion Restaurant, Piccadilly, London, on Thursday, July 1st, 1909.)

Mr. President and Gentlemen,—With your permission, the remarks I shall make now will not take the form of a paper, nor, I am afraid, will they take the form of a very orderly speech. The subject which I have, perhaps unfortunately, chosen is one which would, as you can readily see, occupy a very great deal of time were one systematically to enter, in proper proportion, into all the various parts of it.

So there will be many gaps; upon many points I must touch without my data; I must make many assertions, apparently rash, and I can only ask your indulgence in this sense, that however imperfect the connection may seem to be between the facts and my conclusions, they are the best that I have been able to arrive at during some 40 years of observation and thought on the subject.

I will first trouble you with a few words of ancient history, by way of bringing in my point of view. The late Professor Burdon Sanderson and myself began, under Marey's influence, to work at the sphygmograph, more years ago than I intend now to reckon. Thus we came into association with that very distinguished young physician, only too soon taken from us—Dr. Mahomed of Guy's Hospital. During the early seventies Sanderson and I and Mahomed were working together at this subject, chiefly at the form of the pulse, and then Mahomed started us on the pressures as well as the form, and we were trying by means of the sphygmograph to get some notion of arterial pressures; this I will sum up by saying merely that, to our disappointment, this was not practicable.

Mahomed introduced a phrase which has now almost necessarily fallen into disuse, but which was then common amongst us, namely "the pre-albuminuric stage of Bright's disease." Mahomed was then under the impression, and so were we, that before Bright's disease appeared as a clinical process there was a period, longer or shorter, of elevated blood-pressure, during which time the encroachment of the disease could be foretold.

At that time and at that stage we suffered the very great loss of Dr. Mahomed by death. But, as time went on, I began to find that many of these pre-albuminurics did not pass on into Bright's disease; that on the contrary some of them were cured and got well, which rather shook one's notions on the pre-albuminuric stage of Bright's disease. It was not, however, until I had watched a certain patient for 18 years that I felt at liberty to publish any opinion upon the subject. This lady, who was one of the first to come under my observation as a "pre-albuminuric," never fell a victim to Bright's disease, but lived for 18 years afterwards, during which period she went through a series of morbid processes with high blood-pressure to which I will refer presently, and died ultimately of a lesion which was not Bright's disease in any proper clinical sense of the word; in fact, from my point of view, it was not Bright's disease at all.

Then rather suddenly I left Leeds, I became a Lunacy Commissioner, and visited the asylums, where I found a very large number of persons suffering from arterial disease, yet many of them, I found, somewhat to my surprise, had apparently no rise of blood-pressure. We had at that time got some blood-pressure instruments, and I began to observe that cases should be divided, first, into Bright's disease—which I now put wholly apart and shall not refer to again; and secondly, into the class to which I gave the name Hyperpiesis, a gradual rise of blood-pressure towards middle life or early middle life, which has a course of its own, and deserves the name of a disease; and thirdly, into one other class at least of arterial degeneration which is not typically attended by rise of blood-pressure, in which the blood-pressure does not exceed the rise which is common to all persons when they get on in later life, and of which the symptoms and results are altogether different. At this period I wish to refer to the work which M. Huchard was doing about the same time. A few days ago I fortunately came upon a statement of his latest views by the Paris correspondent of the *Medical Press* of June 2, 1909. M. Huchard's view is that arterial sclerosis is a Disease. At the Toronto meeting I took for my text, as it happened, the very opposite—that arterial sclerosis is not a disease. Arterial sclerosis, I ventured to suggest, is the result of disease; it is the track of disease, but it contains the tracks of several diseases.

Arterial sclerosis may be a result, for instance, of syphilis; or it may be the result of a certain degenerative process going on in the arteries, without necessarily being accompanied by much rise of blood-pressure, and leading more to a calcification of the tunica media; or, again, the result of another kind of disease when the change is more fibrotic, and is an intimal or subintimal process, attended generally by high blood-pressure. Then we have to classify also a large number of toxic kinds of disease which produce arterial sclerosis; diabetes, syphilis, lead poisoning, and so forth. Arterial sclerosis, then, shows where many diseases have passed over, and left, probably not even a pathological unity, but various kinds of arterial disease which are lumped together under this anatomical name. I feel obliged, therefore, while speaking with the greatest respect of the brilliant gifts of our colleague Huchard, to point out where I venture to differ from him at the very onset; and I still stick to my text at

Toronto, that arterial sclerosis is not a clinical name at all; it is an anatomico-pathological result; and it seems to me very misleading to speak of it clinically as A Disease.

I need not follow M. Huchard's description of his disease, but will merely state that he divides it into cardio-renal arterio-sclerosis, cardio-sclerosis of myo-valvular origin, and cardio-valvular disease; the Stokes-Adams type. This is one of those generalisations which leave one breathless; I am very timid in attempting such flights and fear this one will not stand much hard work. How are we possibly to bring a malady like Stokes-Adams disease into any common classification with cases of arterial sclerosis? I must hold to the division which I made in 1894; namely, that we should separate arterial sclerosis first into the process to which I gave the name Hyperpiesis, which does not mean merely high blood-pressure; it means a disease the nature of which is unknown, but which may consist in an absorption of pressor toxins from the intestines. For some reason at any rate, the blood-pressure begins to rise in middle life, at 50 or 55 perhaps, and at first the patient may not feel anything but the better for it; there is a better supply of blood to the brain. But the pressure still rises, and as it does so, whether on account of the toxins themselves, or of the change in the arteries, we cannot tell—the patient feels symptoms which I cannot enter into now, but which are very characteristic, and enable us to make a diagnosis of these cases. This process, if not cured in time, will end in one of various ways—two especially. One is apoplexy, in contrast with the form of arterial sclerosis which I have called the Decrescent or senile form, which does not end in apoplexy, but in an obliteration of the cerebral arteries and thrombosis. To say, as Huchard does, that apoplexy results only from chronic Bright's disease is, again, I think, an impossible position; there are a very large number of cases of apoplexy which belong to Hyperpiesis, which I repeat is not the disease which we call Bright's disease.

Now let us stay to consider another point, the proposal or suggestion that all this hyperpietic disease is primarily of renal origin. This I neither contest nor assert; its primary causes lie in obscurity. What we must recollect is that Bright's disease—and perhaps I had better now confine myself to the chronic "interstitial" form of it—is a certain definite fairly uniform procession of symptoms, attended, I need scarcely say, with high pressures, but also with many other clinical features, such as headache, vomiting, certain diseases of the fundus of the eye; chronic uræmia, passing on, perhaps, into acute uræmia. And we find albumin in the urine generally, and granular casts perhaps always. It is to this definite clinical series to which the name chronic Bright's disease has been given, and in usage is given. But this is not the disease of which I speak under the name Hyperpiesis. It may be asserted that both these diseases have their origin in some renal defect, but such knowledge as we have is rather to the contrary. It is that the disease which I have called Hyperpiesis is due to absorption from the intestines of pressor poisons; such at any rate are the indications of the work done by Prof. Dixon and Dr. Harvey in the Pharmacological Laboratory at Cambridge.

To two statements I must allude about the kidneys in Bright's disease. You all will remember the able essay in which Dr. Batty Shaw described some experiments on the part played by the kidneys in setting up such changes as those I have called Hyperpiesis. Now, whether of recent origin or not, the disease is not Bright's disease: it is another process with a uniform series of symptoms of its own. If you can catch any hyperpietic disease early, say within the first five years, you may cure it, and completely. Or, if it has been going on for four or five years, you may cure it once, but it comes back; you cure it a second time, and the cure is a little more lasting; cure it a third time, and if you are fortunate (which often happens), it never comes back again. Now chronic Bright's disease is never cured. When we speak of the so-called "pre-sclerotic" and "pre-albuminuric" conditions, we are speaking of perfectly curable conditions; not of incipient Bright's disease.

But it may be said the arterio-sclerotic kidney is, or becomes, Chronic Interstitial Nephritis. This I cannot for a moment admit. We know, from the experiments of Rose Bradford and others, how large portions of the kidney may be ablated with impunity; the same is true of other glands in the body, if time is taken to amputate gradually. For the narrower purposes of life there is organ enough left to get on with. So it is with the arterio-sclerotic kidney, a kidney which is very much deformed, lobulated, lumpy, irregular; but is technically not a "granular" kidney, and it should not be called granular. On section you will find in it a large amount of interstitial tissue no doubt, as you would in any organ of an old man; but if you look to the proper tissues of the organ itself, you will find many nests of normal kidney, plenty for an old man to get along with. In life there may have been slight traces of albumen, but few or no casts; and to the end of life the kidneys did well enough. But if the disease begins as chronic Bright's disease, it is a totally different affair. A lady, let us say, is confined; she has albuminuria. If the disorder proceeds, what does it go on to? To chronic Bright's disease. But if she dies in the first and acute stage, what do you find in the kidney? No interstitial changes to speak of. The feature is necrosis of the proper secreting structure of the kidney; widespread necrosis, so complete that she died promptly of uræmia. If instead of dying soon she goes on longer; if she lives even ten years, yet in the beginning and in nature the process was the same, a necrotic, destructive process in the proper secreting tissue of the kidney to which the fibrosis is secondary. This is not the arterio-sclerotic kidney; nor is it an old man's substitution-fibrosis; it is the ultimate consequence of a poison which destroys the epithelial tubes at their origin. Every case of Bright's disease consists in an attack upon the proper secreting structure of the kidney. The arterio-sclerotic fibrous kidney is in its very nature to be divided from that of chronic Bright's disease.

I need not delay you to say the same things of the pancreas; and I think the emphysema of the lungs, which nearly always occurs in these cases, is also of the same nature.

I have spoken of apoplexy as one fatal termination of Hyperpiesis. Another frequent termination is by cardiac defeat rather than by cardiac failure. We hear every day now, that So-and-so died of "cardiac failure"; it is the fashionable phrase; as in our childhood we were puzzled with the question, "What did So-and-so die of?" the answer being "Shortness of breath." The modern Mr. So-and-so dies of "cardiac failure." I find in consultations also that the fault is always laid at the door of this poor organ, as having dilated and given way, degenerated, become sclerosed, etc.; whereas, on the contrary, the truth is that in cases of Hyperpiesis the heart gradually rises in value; it becomes largely hypertrophied, contains more blood, and drives that blood out with extraordinary power at pressures of 180, 200 220, 240 millimetres, and this for 5, 10, or 15 years. If ultimately it is defeated, is it fair to call its defeat a failure—except, perhaps, in a very temporary sense?

Here is an unhappy organ, overworking itself, developing an enormous power, 50 per cent. perhaps, above normal duty, for a number of years, and when ultimately it is defeated people talk of cardiac failure, and M. Huchard accuses it of degenerating in common with its arteries. Is it reasonable thus to disparage the heart which can no longer carry out an enormous task? In most cases Hyperpiesis is either cut short by apoplexy, or, if fought out to the bitter end, the heart dies not of any intrinsic vice or defect, but bravely in defeat.

I do not know why we talk of "cardio-sclerosis" at all; I do not know precisely what is meant by it. I know that in old people's hearts we find fibrous tissue more or less mixed with true muscular tissue, as we find in any other organ of the body, including the skin. But why stigmatise it as cardio-sclerosis? These hearts are enormously strong; they are doing an enormous quantity of work. Their elasticity is within narrower limits, but it is very high. I do not think we should look upon this state as cardiac disease. The coronary arteries are often diseased it is true. But Prof. Kanthack and I collected all the hearts we could—fresh ones—in cases of coronary sclerosis (the hypertrophic type), examined those hearts carefully, and concluded that the relation between the heart degeneration and the occlusion of the coronary arteries was chiefly a time relation. If the coronary arteries were blocked up rapidly, rapid and considerable cardiac degeneration ensued. But if the change extended over a very long period of time, although the coronary arteries were absolutely occluded, so that you could not say where they had been, you might and often did find a very fairly normal heart; sclerotic in the sense of having more common fibre than usual no doubt, but powerful enough to have driven blood through aortic orifices often of extreme stenosis. How can we call such hearts diseased in a clinical sense? Such a heart may have driven blood with prodigious velocity through an opening which would scarcely admit a probe; although the closed coronary mouths were barely visible. The alternative circulation probably is by development of the veins of Thebesius. Apart from toxic influences, the resources and readaptive capacities of the heart are amazing.

Thus I have divided arterial sclerosis into three kinds—into (i) Hyperpiesis, which is a disease, and not merely a play of the blood pressures; (ii) a Toxic form, which I am sure you may well hope I shall not enter upon now, a state caused by syphilis, diabetes, etc., in some cases with high pressure—lead has it, diabetes has not. You may see arterial sclerosis in diabetic children, with a blood pressure of 90 or 100; and so again in phthisis, or syphilis. And (iii) there is the Decrescent form, which is a medial rather than an intimal disease, and is not attended with notably high blood pressures.

In some hyperpietic cases you find the radial artery very closely contracted, in other cases it is large and leathery; and I suppose in the contracted cases the peripheral constriction, which we assume in all these cases, extends very much higher up in the arteries of tertiary magnitude; and, as a matter of experience, the cases with the small contracted radials are very intractable, and are generally fatal by way of apoplexy.

In the Decrescent form people live to very long ages, and some of them are not very much the worse for this degeneration; although their functional capacity must be reduced. Yet I saw a gentleman not long ago, about 70 years of age, whose arteries are perfectly grotesque, thick and curly; yet he is very healthy, very well and active; and one day I surprised him in a game of tug-of-war with eight or ten junior friends. You may go into any hospital or workhouse and see quantities of such arterial sclerosis in people who live to great ages. The danger in this form is lest cerebral arteries silt up, and the brain suffer accordingly; but this is a different event from hyperpietic apoplexy.

It was the view of v. Basch, to whom we all owe so great a debt, that the high blood pressure is the result of the arterial change. But how can this be when we see these people, with blood pressures not more than 150, living to old age, and yet with the most contorted arteries? It is alleged that they must have high blood pressure because their areas of irrigation are shrivelled, therefore the blood not having the same space to occupy, the blood pressure must rise. This is virtually the old story of blood pressures depending on blood mass.

Mere loss of area has nothing to do with pressures. To make any effect on the blood pressure, the difference of blood mass or alternately of area of irrigation would have to be far more than ever comes to pass. Pressures can rise only when very large areas are constricted universally and together.

Before we can formulate any reasonable principles of treatment we must attain to clearer notions of the several processes concerned; in this purpose I have been endeavouring to assist. How these notions help us to a rational therapeutics I have explained in former papers, and hope before long to explain still more at length, but this evening I have detained you already too long.

